# DNA fragmentation in some organs of rats and mice treated with cycasin.

**DOI:** 10.1038/bjc.1979.70

**Published:** 1979-04

**Authors:** M. Cavanna, S. Parodi, M. Taningher, C. Bolognesi, L. Sciabà, G. Brambilla

## Abstract

Cycasin (methylazoxymethanol-beta-D-glucoside) is carcinogenic in several animal species. It produces a variety of malignant tumours, mainly in the liver of mice, and in the liver, kidney and large intestine in rats. It does not appear to be mutagenic in the Ames test, even in the presence of liver microsome fraction, and it is among those carcinogens (less than 10%) ranked as "false negatives" in this test. The ability of cycasin to damage in vivo liver, kidney, lung and colonic DNA of Wistar rats and C57BL/L mice was investigated by means of alkaline elution technique. Oral single-dose administration of cycasin, in the range of 50-400 mg/kg body weight, produced in the rat a clearly evident dose-dependent DNA fragmentation in the liver, and less marked damage to DNA from kidney and colon mucosa. In mice, the same treatment produced dose-dependent DNA damage only in the liver. DNA repair up to 18 h appeared to be incomplete both in mice and rats. Methylazoxymethanol acetate is considered to be an active form of cycasin. While in vivo methylazoxymethanol acetate caused DNA damage, in vitro it appeared inactive and required metabolic activation, possibly consisting in its hydrolysis by esterase activity, to be able to cause DNA fragmentation.


*Br. J. Cancer* (1979) **39**, 383

### DNA FRAGMENTATION IN SOME ORGANS OF RATS AND MICE TREATED WITH CYCASIN

M. CAVANNA,* S. PARODI,† M. TANINGHER,† C. BOLOGNESI,† L. SCIABÀ* AND G. BRAMBILLA*

*From the Departments of *Pharmacology and †Oncology, University of Genoa, I-16132 Genoa, Italy*

Received 7 August 1978 Accepted 3 January 1979

**Summary.—Cycasin (methylazoxymethanol-β-D-glucoside) is carcinogenic in several animal species. It produces a variety of malignant tumours, mainly in the liver of mice, and in the liver, kidney and large intestine in rats. It does not appear to be mutagenic in the Ames test, even in the presence of liver microsome fraction, and it is among those carcinogens (less than 10%) ranked as "false negatives" in this test. The ability of cycasin to damage *in vivo* liver, kidney, lung and colonic DNA of Wistar rats and C57BL/6 mice was investigated by means of alkaline elution technique. Oral single-dose administration of cycasin, in the range of 50-400 mg/kg body weight, produced in the rat a clearly evident dose-dependent DNA fragmentation in the liver, and less marked damage to DNA from kidney and colon mucosa. In mice, the same treatment produced dose-dependent DNA damage only in the liver. DNA repair up to 18 h appeared to be incomplete both in mice and rats. Methylazoxymethanol acetate is considered to be an active form of cycasin. While *in vivo* methylazoxymethanol acetate caused DNA damage, *in vitro* it appeared inactive and required metabolic activation, possibly consisting in its hydrolysis by esterase activity, to be able to cause DNA fragmentation.**

CYCASIN (methylazoxymethanol-β-D-glucoside), which occurs in cycad plants used by some populations as food, has been reported to be carcinogenic in rodents (mice, rats, hamsters, guinea-pigs and rabbits) (IARC, 1976). Unlike several other carcinogens, the ability of cycasin to induce DNA damage and/or repair, as evaluated by the appearance of single-strand breaks or weak points in alkali, has not yet been studied. This work was therefore undertaken to study DNA damage and its repair in some organs of mice and rats given single oral doses of cycasin, and to examine whether a correlation exists between the organotropism of carcinogenic activity and the extent of DNA fragmentation. A comparison between cycasin and methylazoxymethanol acetate was made to determine whether the latter agent also requires metabolic activation. A new method for detecting DNA damage *in vivo* by the use of alkaline elution has been used (Parodi *et al.*, 1978; Brambilla *et al.*, 1978). A ranking tabulation of dose-related DNA damage induced by cycasin in rats has been preliminarily reported (Parodi *et al.*, 1978).

### MATERIALS AND METHODS

In these experiments C57BL/6 male mice and Wistar male rats, aged 1–2 months, were used. Cycasin (a gift from Dr H. Matsumoto, University of Hawaii at Manoa, Honolulu) was dissolved in distilled water and administered orally by gastric intubation at varying single-dose levels and intervals before animals were killed by cervical dislocation and exsanguinated. Methylazoxymethanol acetate (ICN Pharmaceuticals, Inc., Plainview, New York, U.S.A.) was dissolved in physiological saline and injected i.p. into C57BL/6 male mice. Liver, kidneys, lungs and descending colon were quickly removed and rinsed in cold Merchant's solution (140mM NaCl, 1·47mM $KH_2PO_4$, 8·1mM $Na_2HPO_4$, 2·7mM KCl, 0·53mM $Na_2EDTA$, pH 7·4). Liver and kidneys were minced in Merchant's solution and homogenized by using a loose-fitting Potter-Elvehjem homogenizer. Small fragments of lungs and colon mucosa were minced in Merchant's solution and then forced through a stainless-steel screen (No. 60 mesh). After sedimentation of the larger fragments in any tissue suspension, the crude cell suspension in the supernatant was pelleted at 50 *g* for 4 min, and resuspended in a suitable volume of the above solution for a counting in a haemocytometer.

The alkaline elution was essentially carried out accordingly to Kohn *et al.* (1976) with the modification previously described (Parodi *et al.*, 1978; Brambilla *et al.*, 1978). About $10^6$ cells were loaded on to a millipore filter made of mixed esters of cellulose (25 mm diameter, 5 $\mu$m pore size), and washed with cold Merchant's solution. The cells were lysed on the filter at room temperature by passing 4·5 ml of 0·2% sodium N-lauroyl sarcosinate, 2M NaCl, 20mM $Na_2EDTA$, pH 10·2. The filter was then washed with 3 ml of 20mM $Na_2EDTA$, pH 10·2. Single-stranded DNA was eluted from the filter in the dark with 20 ml of a solution consisting of 60mM tetraethylammonium hydroxide and 20mM $Na_2EDTA$, pH 12·3, pulled at a peristaltic pump speed of 0·2 ml/min. Fractions were collected every 10 min. The filter was then placed in 2 ml of eluting solution and broken up with a blender (filter fraction); the system without the filter membrane was washed with 2 ml of eluting solution (washing fraction). DNA content of any fraction was determined according to the following modification (Parodi *et al.*, 1978; Brambilla *et al.*, 1977, 1978) of the microfluorometric technique of Kissane & Robins (1958). The DNA from each fraction was precipitated with trichloroacetic acid and washed with absolute ethanol. The pellets were air-dried at room temperature. A volume of 0·03 ml of an aqueous solution of 3,5-diaminobenzoic acid dihydrochloride (400 mg/ml) was added to each sample and, after mixing, the tubes were incubated for 30 min at 70°C. After cooling, 1·5 ml of 0·6N perchloric acid was added to each tube. The fluorescence was read at 520 nm with an excitation wavelength of 436 nm (OB 10 filter) in a EEL 244 fluorimeter. The blank readings were made from tubes containing 1 ml of eluting solution subjected to the same procedure. The fraction of total fluorescence was determined for each of the collected fractions. The rate of DNA elution in the initial phase was describable by first-order kinetics and dependent directly on the dose of the DNA-damaging agent (Kohn *et al.*, 1976). The rate constant, $K$, can be considered as an inverse measure of DNA single-strand size (Kohn *et al.*, 1976). Our results, therefore, will be expressed as initial rate constant of DNA elution with respect to the eluted volume, and such a value, $K$ ($ml^{-1}$), has been obtained from the following equation:

$$Q = Q_0 \cdot e^{-Kv}$$

where $Q_0$ (=1) is the amount of DNA stored on the filter before elution starts (zero elution volume), and $Q$ is the fraction of DNA retained on the filter at the $v$ elution volume in ml.

To evaluate the DNA-damaging activity of methylazoxymethanol, the supposed ultimate electrophilic reactant of cycasin, some experiments were carried out on EUE cells, a human heteroploid line isolated by Terni & Lo Monaco (1958), obtained from Istituto Mario Negri, Milan, Italy. These cells, free from mycoplasm contamination, were grown in 8 $cm^2$ Leighton tubes in the presence of 2·5 ml of Dulbecco's modification of Eagle's minimal essential medium supplemented with 10% calf serum and 5% foetal bovine serum. The cells were challenged for 1 h with methylazoxymethanol acetate, with mouse

TABLE.—*Initial rate constant of elution, K (ml⁻¹), of DNA from EUE cells exposed to methylazoxymethanol acetate (MAM ac.), C57BL/6 mouse liver supernatant (LS), and acetic acid, for 1 h*

| Treatment | | | $K$ ($ml^{-1}$) values | |
|---|---|---|---|---|
| MAM ac. (mg/ml) | LS (%) | Acetic acid (mg/ml) | Mean | (range) |
| 1 | — | — | 0·013 | (0·01–0·02) |
| 2 | — | — | 0·020 | (0·02–0·02) |
| 1 | 10 | — | 0·270 | (0·21–0·33) |
| — | 10 | — | 0·020 | (0·02–0·02) |
| — | 10 | 0·45 | 0·025 | (0·01–0·05) |
| — | — | 0·45 | 0·040 | (0·03–0·05) |
| — | — | — | 0·015 | (0·01–0·03) |

The means were calculated from at least 2 values, each of them being obtained from one cell culture in an individual Leighton tube.

liver supernatant, or with acetic acid, as indicated in the Table. The cells were then washed and detached with cold Merchant's solution, without any chase time. About $5 \times 10^5$ cells were loaded on to the filter for alkaline elution as previously described. The supernatant of liver cells was obtained by homogenizing the liver from C57BL/6 male mice with 9 volumes of cold Merchant's solution in a Potter-Elvehjem homogenizer, and by centrifuging the homogenate at 1000 *g* for 10 min.

### RESULTS

The following data demonstrate the capability of cycasin to damage liver DNA in mice, and the DNA of liver kidney, and colon mucosa in rats. Not having identified the type of damage, we will consider increased elution rate of DNA following cycasin administration as DNA damage, and the return with the time of the DNA elution rate toward the control level as repair of the damage.

Fig. 1 shows the elution rates, expressed as $K$ (ml$^{-1}$), of DNA from liver, lungs, kidneys and colon mucosa of rats killed 4 h after oral treatment with 50, 100, 200 or 400 mg/kg body weight of cycasin. A differential damaging effect on DNA of the 4 tissues was found, liver being the most sensitive. A dose-dependent increase in DNA elution rate was evident in the entire dose range that was tested. Kidney and colon mucosa were markedly less sensitive. Definite DNA damage occurred only at the doses of 200 and 400 mg/kg. The lung appeared insensitive at all dosages. In mice killed 6 h after oral administration of

FIG. 1.—Dependence on cycasin dose of the initial rate constant of elution, $K$ (ml$^{-1}$), of DNA from liver, colon, kidney and lung of Wistar rats killed 4 h after treatment by oral route. Each reported value represents the mean of at least 2 experiments. The 13 control values, accumulated for all the organs, have a mean of 0·030 ± 0·012 (s.d.) Where the range bar (maximum–minimum value interval) is not represented, the single values fall within the range of 3 s.d. from the mean of the accumulated control values.

FIG. 2.—Dependence on cycasin dose of the initial rate constant of elution, $K$ (ml$^{-1}$), of DNA from liver, kidney, colon and lung of C57BL/6 mice killed 6 h after treatment by oral route. Initial rate constant of elution of DNA from the liver of C57BL/6 mice killed 4 h after i.p. administration of methylazoxymethanol (MAM) acetate (50 mg/kg body weight) and of control mice are also recorded. Each reported value represents the mean of at least 2 experiments. The 20 control values, accumulated for all the organs, have a mean of $0{\cdot}026 \pm 0{\cdot}011$ (s.d.). Where the range bar (maximum–minimum value interval) is not represented, the single values fall within the range of 3 s.d. from the mean of the accumulated control values.

cycasin (Fig. 2) only liver DNA showed a dose-dependent increase in elution rates for doses ranging from 50–400 mg/kg, while in the other organs there was no definite modification of DNA elution pattern. In mice killed 4 h after i.p. administration of 50 mg/kg body weight of methylazoxymethanol acetate, the increase of liver DNA elution rate was sharp, and close to that obtained 6 h after the administration of 200–400 mg/kg of cycasin by oral route (Fig. 2).

The time sequence to a fixed dose of cycasin in terms of initial elution rate of DNA from the various organs from rats and mice was examined. This will produce evidence either of the interval between cycasin administration and maximum DNA damage, or of the duration of its repair. In rats (Fig. 3) the maximum effect was seen by 4 h for the liver, by 2–4 h for the kidney, by 4–6 h for the colon mucosa, whereas there was no effect at any time for the lung. Within the limits of variability of our results, the DNA repair appeared more evident in rat liver than in kidney and colon in the 4–12 h interval. The high level of rat liver DNA damage observed by 4 h, after 200 mg/kg body weight of cycasin, seems to be corroborated by the evidence of the dose-dependence of such a damage at the same interval, as shown in Fig. 1. In mice (Fig. 4) the maximum effect was seen by 6–12 h for liver, whereas there was practically no effect at any time for the other 3 organs. Little, if any, repair was seen by 4–18 h in the liver. Further investigation would be

FIG. 3.—Dependence on interval after treatment of initial rate constant of elution, $K$ (ml$^{-1}$), of DNA from liver, kidney, colon and lung of Wistar rats given 200 mg/kg of cycasin by oral route. Each reported value represents the mean of at least 2 experiments. The 13 control values, accumulated for all the organs, have a mean of $0{\cdot}030 \pm 0{\cdot}012$ (s.d.). Where the range bar (maximum–minimum value interval) is not represented, the single values fall within the range of 3 s.d. from the mean of accumulated control values.

needed to assess better whether there is a real difference in the kinetics of liver DNA repair between the two species.

Histological examination of liver, lung, kidney and colon mucosa from animals killed 24 h after administration of cycasin 200 mg/kg body weight did not furnish any evidence of cellular necrosis.

EUE cells exposed to 1 and 2 mg/ml methylazoxymethanol acetate for 1 h displayed DNA elution rates without evidence of single-strand breaks. The $K$ (ml$^{-1}$) values appeared throughout superimposable for both control and treated cells (Table). However, in the presence of liver supernatant, which could release methylazoxymethanol from methylazoxymethanol acetate by esterase activity, methylazoxymethanol acetate abruptly increased the DNA elution rate. Either liver supernatant, or a stoichiometric concentration of acetic acid in respect to methylazoxymethanol acetate, or both together, produced no effects (Table). The hydrolysis of methylazoxymethanol acetate in the presence of liver supernatant was suggested by a shift in pH of ~1·5 units (from 7·2 to 5·7). A shift in pH of the same order was obtained when acetic acid alone was added stoichiometrically to the concentration of methylazoxymethanol acetate. In our experimental conditions, the incubation system appeared to be very rich in esterase activity, because, at 37°C, less than 3 min was required to complete the pH shift.

### DISCUSSION

Experimental evidence indicates that cycasin is a peculiar type of procarcinogen depending on a glucosidase for its activation to the biologically active aglycone methylazoxymethanol. In fact, cycasin is

FIG. 4.—Dependence on interval after treatment of initial rate constant of elution, $K$ (ml$^{-1}$), of DNA from liver, colon, kidney and lung of C57BL/6 mice given 200 mg/kg of cycasin by oral route. Each reported value represents the mean of at least 2 experiments. The 20 control values, accumulated for all the organs, have a mean of $0{\cdot}026 \pm 0{\cdot}011$ (s.d.). Where the range bar (maximum–minimum value interval) is not represented, the single values fall within the range of 3 s.d. from the mean of accumulated control values.

carcinogenic in adult rodents only by oral route (IARC, 1976) because of the presence in the gut of a $\beta$-D-glucosidase from the enteric bacterial flora (Kobayashi & Matsumoto, 1965; Laqueur & Spatz, 1968) whilst its carcinogenic activity by s.c. injection is restricted to the early postnatal period, due to the transient presence in newborns of a glucosidase in the subcutaneous tissue (Spatz, 1968; Shibuya & Hirono, 1973). Moreover, cycasin, because of the glucosidase requirement for its activation, is non-mutagenic in the Ames *Salmonella* test even in the presence of liver microsomal fraction (Smith, 1966; McCann *et al.*, 1975; McCann & Ames, 1976) but is mutagenic in the host-mediated assay (Gabridge *et al.*, 1969). On the contrary, methylazoxymethanol and its synthetic ester methylazoxymethanol acetate were shown to be carcinogenic in adult rats and hamsters (IARC, 1976) also by the parenteral route, and methylazoxymethanol without microsomal fraction was mutagenic in bacteria on Petri dishes (Smith, 1966). Furthermore, evidence of methylation at the N-7 position of guanine has been reported for methylazoxymethanol *in vitro* (Matsumoto & Higa, 1966) and for cycasin and methylazoxymethanol acetate *in vivo* (Shank & Magee, 1967; Nagata & Matsumoto, 1969).

Damjanov *et al.* (1973) observed, by the use of alkaline sucrose-gradient sedimentation, slowly reparable single-strand breaks in DNA from liver of Wistar rats treated i.p. with methylazoxymethanol acetate, but no experiments with cycasin were reported. On the contrary, Van Den Bergh (1974) did not see *in vitro* any reduction in the size of DNA from HeLa $S_3$ cells exposed for 1 h to 0·5 mg/ml of methylazoxymethanol acetate, although the treatment inhibited the ligation of DNA replicons, noticeable with increasing chase time. Because of these apparently contradictory findings about what is considered to be an active form of cycasin, we tried to verify them by the application of alkaline elution. *In vivo* we observed DNA damage in the liver of C57BL/6 mice treated i.p. with methylazoxymethanol acetate, and *in vitro* we found no evidence of alteration in DNA from EUE cells exposed for 1 h to high concentrations of this agent. However, in the presence of liver supernatant, methylazoxymethanol acetate produced a prompt increase in DNA elution rate, probably because of hydrolysis in methylazoxymethanol and acetic acid, after which methylazoxymethanol can work as an ultimate carcinogen. A stoichiometric concentration of acetic acid, with or without liver supernatant, appeared ineffective. Therefore, methylazoxymethanol acetate seems to require hydrolysis, readily obtainable *in vivo*, but *in vitro* only in the presence of esterase activity. Our results demonstrate that the administration in the rat of single oral doses of cycasin produced a clearly evident dose-dependent damage of liver DNA, which agrees with the previously observed (Shank & Magee, 1967) methylation of liver DNA in rats given cycasin by stomach tube. A markedly lower, but still clearly evident, damage was present in DNA of kidney and colon mucosa, the lung DNA being unaffected by doses of cycasin up to 400 mg/kg. In the mouse, in the same experimental conditions, DNA fragmentation is restricted to the liver.

This experimental evidence indicates that the alkaline elution technique allows the rapid evaluation of the DNA damage exerted *in vivo* by a carcinogen-like cycasin, which does not depend for its metabolic activation to a reactive intermediate on the microsomal metabolizing system. Such an ability to reveal the DNA damage, induced *in vivo* by compounds possessing a peculiar type of metabolic activation, has been assessed with 1,2-dimethylydrazine (Brambilla *et al.*, 1978) another carcinogen which gives false negative results with the Ames *Salmonella*/microsome mutagenicity test (McCann *et al.*, 1975; McCann & Ames, 1976).

It is evident *a priori* that the present method, in terms of metabolic activation, has the same flexibility as carcinogenicity tests, in the sense that all the different capabilities of metabolic activation of the various tissues can be exploited. A quantitative comparison of our results with carcinogenicity data is precluded, since these data were obtained with animals of various strains and age, with different dosage schedules and routes of administration. However, results obtained with cycasin show slowly repairable DNA damage in those organs which are the main targets of its carcinogenic activity: liver, kidney and intestine in the rat, and liver in the mouse (IARC, 1976).

This work was supported in part by a grant from the Consiglio Nazionale delle Ricerche, Rome.

### REFERENCES

BRAMBILLA, G., CAVANNA, M., SCIABA, L., CARLO, P., PARODI, S. & TANINGHER, M. (1977) A procedure for the assay of DNA damage in mammalian cells by alkaline elution and microfluorometric DNA determination. *Ital. J. Biochem.*, **26**, 419.

BRAMBILLA, G., CAVANNA, M., PARODI, S., SCIABA, L., PINO, A. & ROBBIANO, L. (1978) DNA damage in liver, colon, stomach, lung and kidney of BALB/c mice treated with 1,2-dimethylhydrazine. *Int. J. Cancer*, **22**, 174.

DAMJANOV, I., COX, R., SARMA, D. S. R. & FARBER, E. (1973) Patterns of damage and repair of liver DNA induced by carcinogenic methylating agents *in vivo*. *Cancer Res.*, **33**, 2122.

GABRIDGE, M. G., DENUNZIO, A. & LEGATOR, M. S. (1969) Cycasin: detection of associated mutagenic activity *in vivo*. *Science*, **163**, 689.

IARC (1976) *IARC Monographs on the Evaluation of Carcinogenic Risk of Chemicals to Man*. Vol. 10. pp 121–138. Lyon: International Agency for Research on Cancer.

KISSANE, J. M. & ROBINS, E. (1958) The fluorometric measurement of deoxyribonucleic acid in animal tissue with special reference to the central nervous system. *J. Biol. Chem.*, **233**, 184.

KOBAYASHI, A. & MATSUMOTO, H. (1965) Studies on methylazoxy methanol, the aglycone of cycasin. *Arch. Biochem.*, **110**, 373.

KOHN, K. W., ERICKSON, L. C., EWIG, R. A. G. & FRIEDMAN, C. A. (1976) Fractionation of DNA from mammalian cells by alkaline elution. *Biochemistry*, **15**, 4629.

LAQUEUR, G. L. & SPATZ, M. (1968) Toxicology of cycasin. *Cancer Res.*, **28**, 2262.

MATSUMOTO, H. & HIGA, H. H. (1966) Studies on methylazoxymethanol, the aglycone of cycasin: methylation of nucleic acids *in vitro*. *Biochem. J.*, **98**, 20C.

MCCANN, J., CHOI, E., YAMASAKI, E. & AMES, B. N. (1975) Detection of carcinogens as mutagens in the Salmonella microsome test: Assay of 300 chemicals. *Proc. Natl Acad. Sci. USA*, **72**, 5135.

McCann, J. & Ames, B. N. (1976) Detection of carcinogens as mutagens in the *Salmonella*/ microsome test: Assay of 300 chemicals: Discussion. *Proc. Natl Acad. Sci. USA*, **73**, 950.

Nagata, Y. & Matsumoto, H. (1969) Studies on methylazoxymethanol: methylation of nucleic acids in the fetal rat brain. *Proc. Soc. Exp. Biol. Med.*, **132**, 383.

Parodi, S., Taningher, M., Santi, L., Cavanna, M., Sciaba, L., Maura, A. & Brambilla, G. (1978) A practical procedure for testing DNA damage *in vivo* proposed for a prescreening of chemical carcinogens. *Mutat. Res.*, **54**, 39.

Shank, R. C. & Magee, P. N. (1967) Similarities between the biochemical actions of cycasin and dimethylnitrosamine. *Biochem. J.*, **105**, 521.

Shibuya, C. & Hirono, I. (1973) Relations between postnatal days of mice and carcinogenic effect of cycasin. *Gann*, **64**, 109.

Smith, D. W. E. (1966) Mutagenicity of cycasin aglycone (methylazoxymethanol), a naturally occurring carcinogen. *Science*, **152**, 1253.

Spatz, M. (1968) Hydrolysis of cycasin by β-D-glucosidase in skin of newborn rats. *Proc. Soc. Exp. Biol. Med.*, **128**, 1005.

Terni, M. & Lo Monaco, G. B. (1958) Coltura continua di cellule derivate da embrione umano. *Sperimentale*, **108**, 177.

Van Den Berg, H. W. (1974) Alkaline sucrose gradient sedimentation studies of DNA from HeLa $S_3$ cells exposed to methylmethanesulphonate or methylazoxymethanol acetate. *Biochim. Biophys. Acta*, **353**, 215.


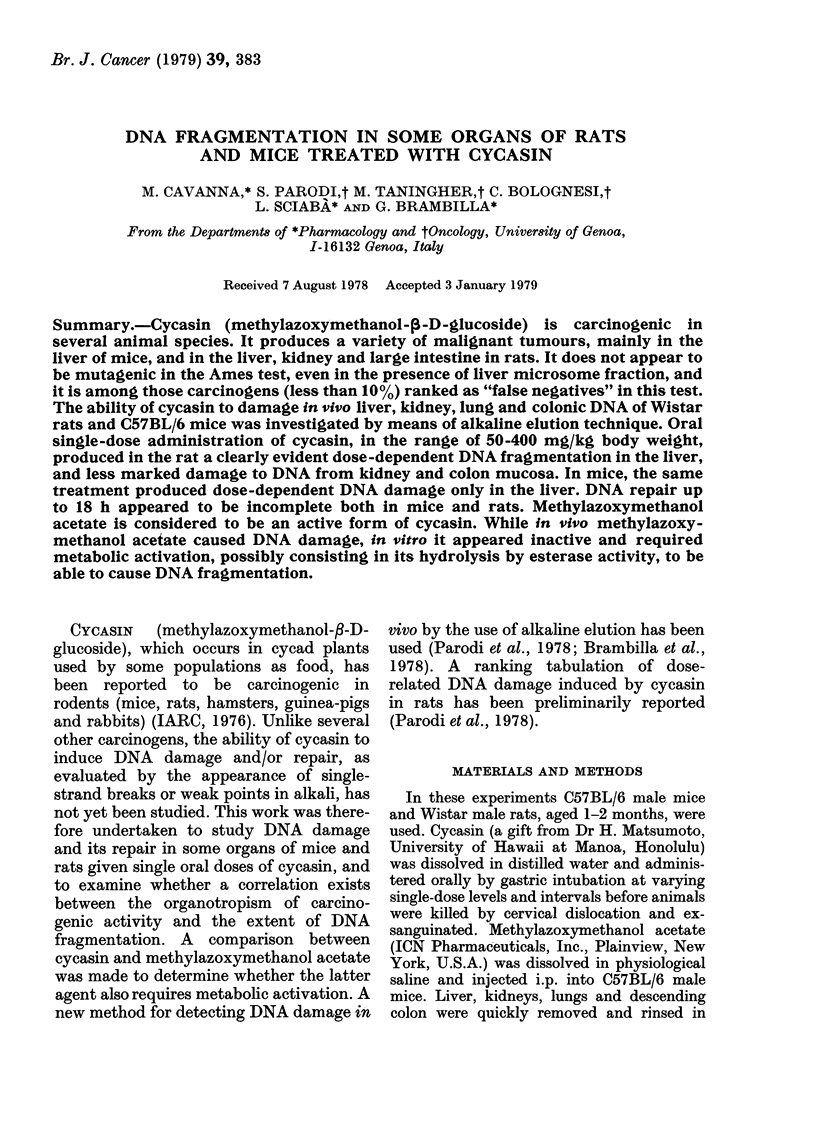

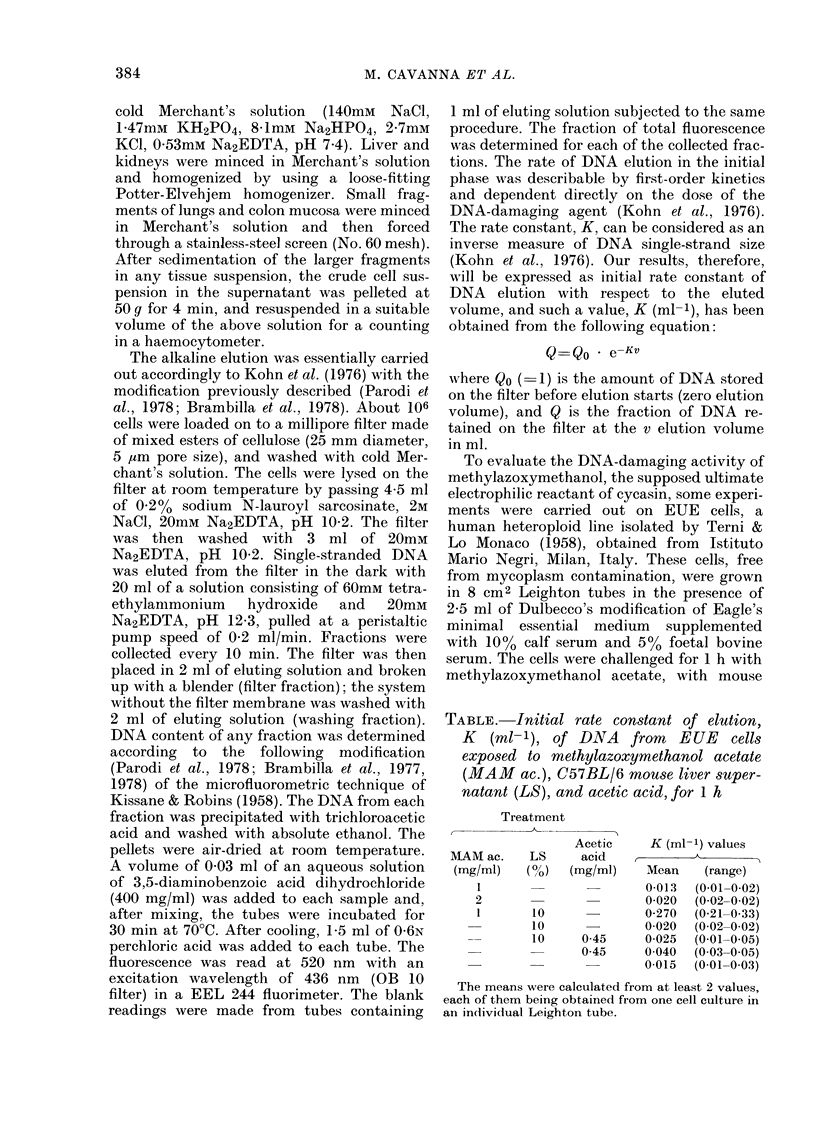

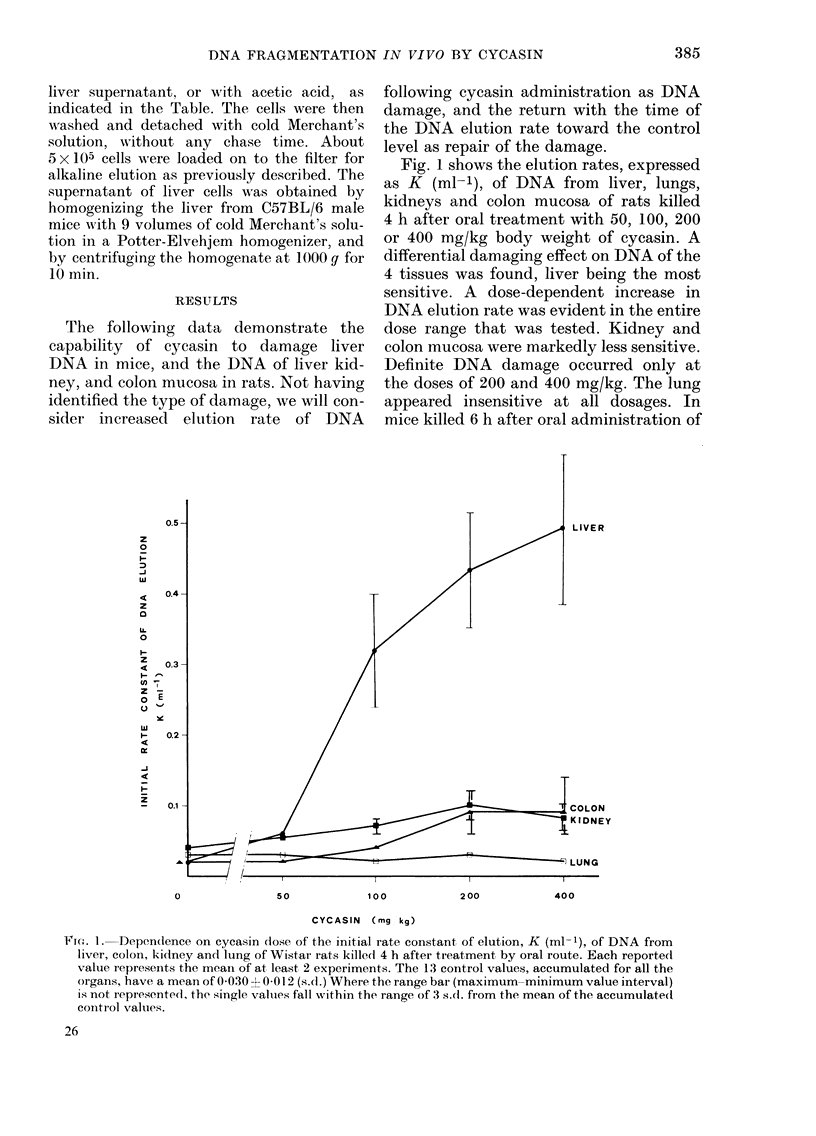

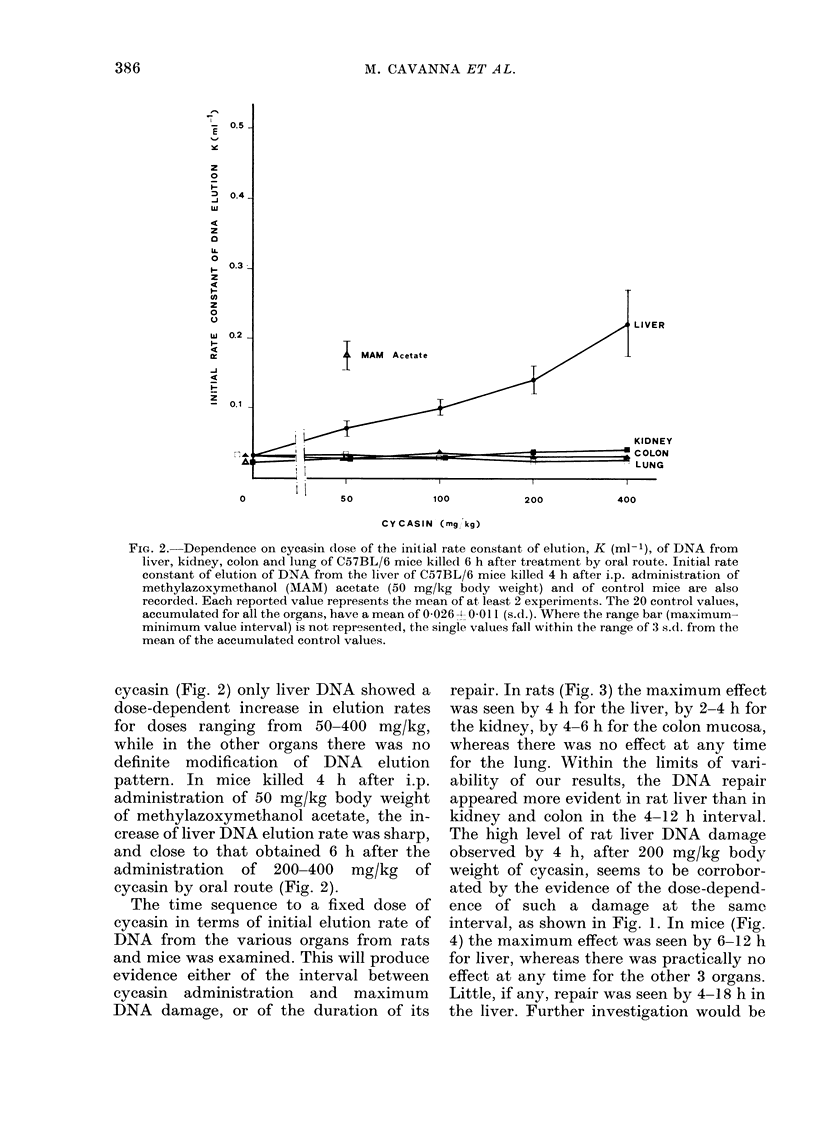

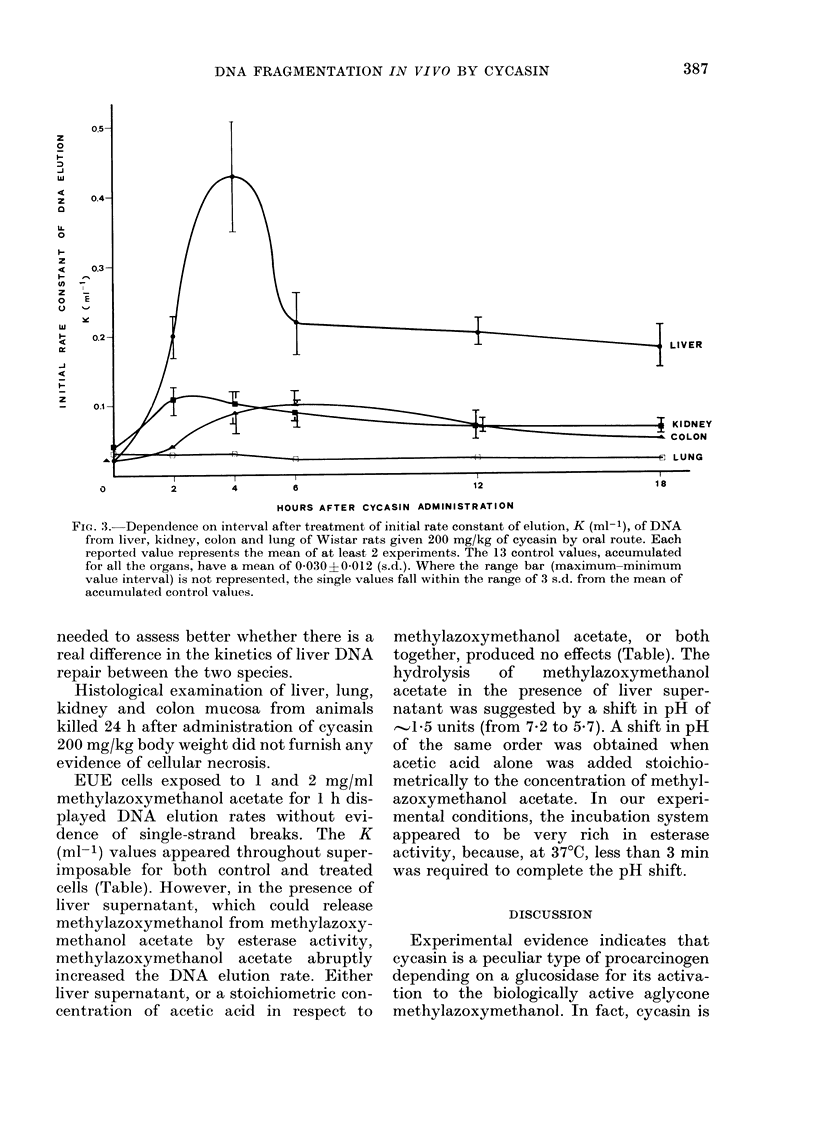

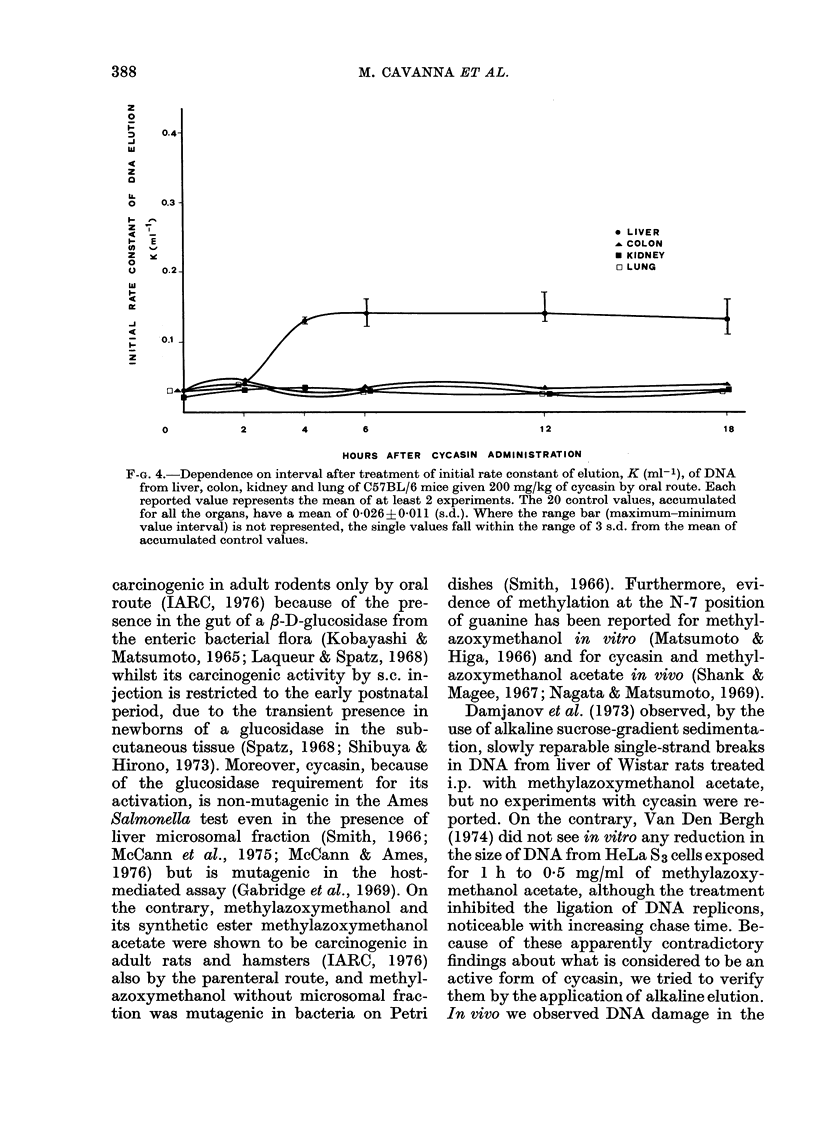

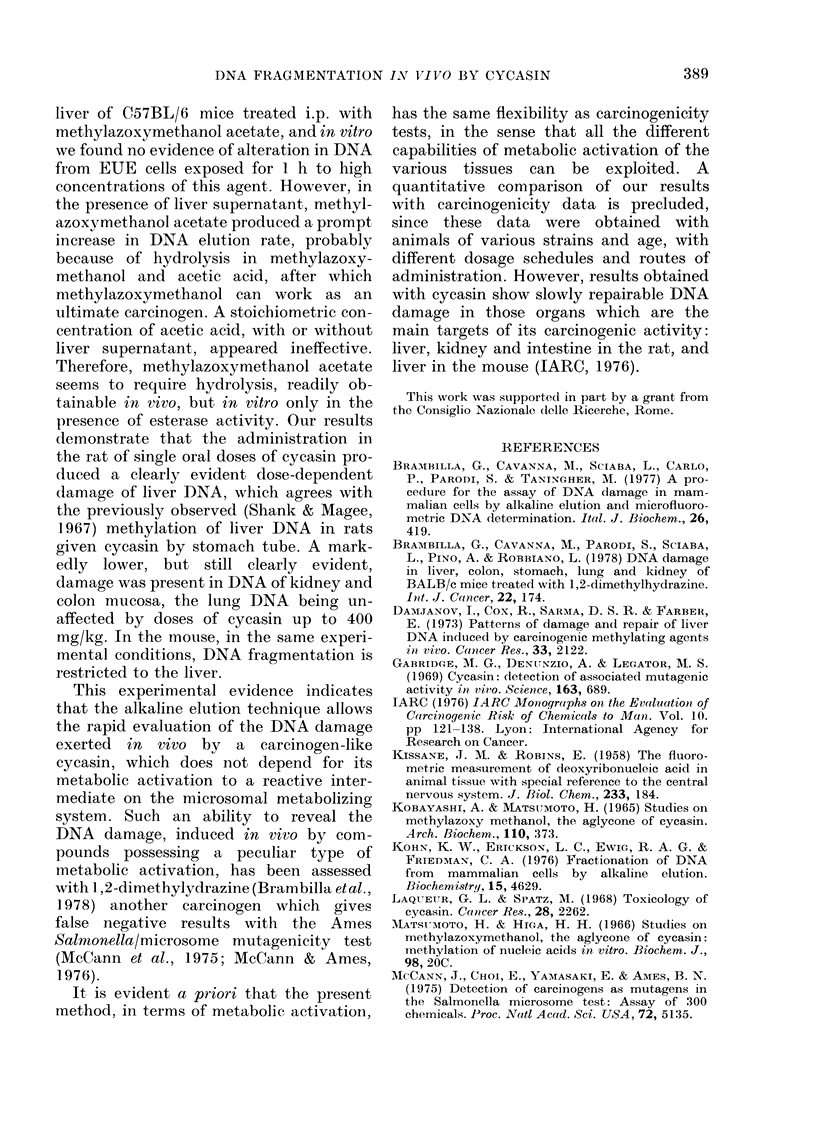

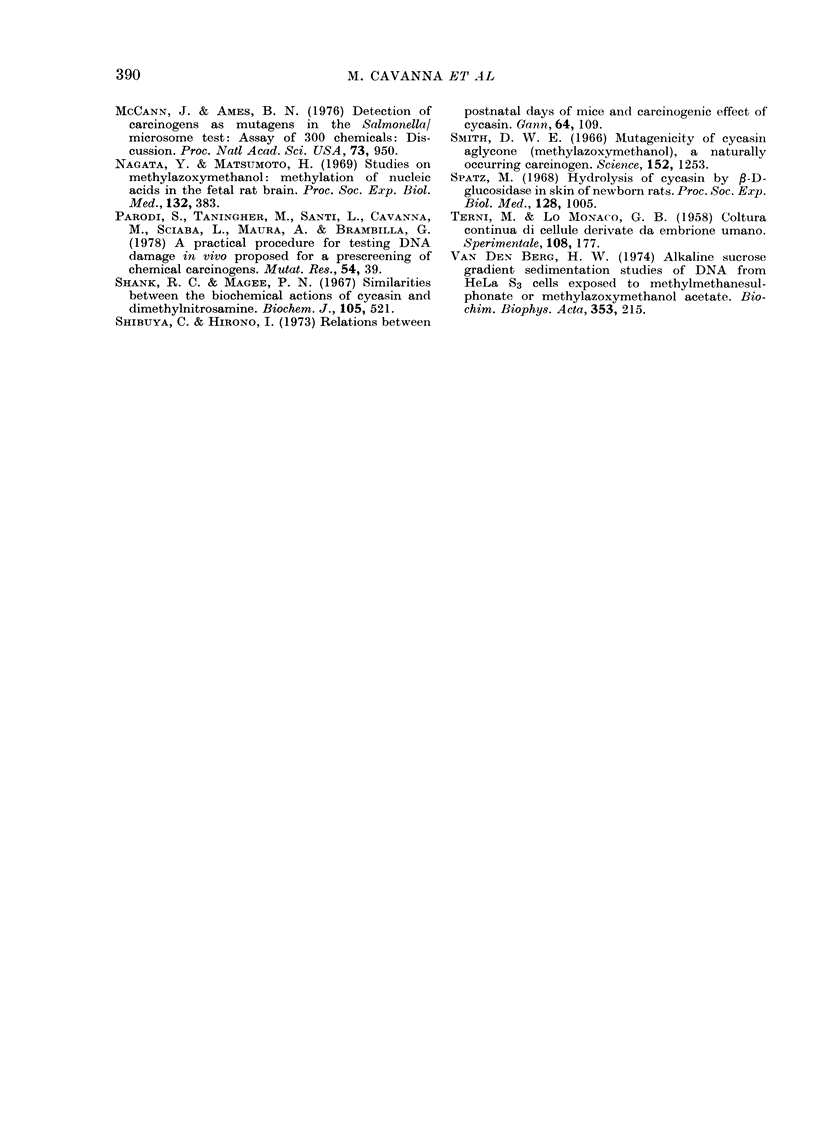


## References

[OCR_00765] Brambilla G., Cavanna M., Parodi S., Sciaba L., Pino A., Robbiano L. (1978). DNA damage in liver, colon, stomach, lung and kidney of BALB/c mice treated with 1,2-dimethylhydrazine.. Int J Cancer.

[OCR_00757] Brambilla G., Cavanna M., Sciabà L., Carlo P., Parodi S., Taningher M. (1977). A procedure for the assay of DNA damage in mammalian cells by alkaline elution and microfluorometric DNA determination.. Ital J Biochem.

[OCR_00772] Damjanov I., Cox R., Sarma D. S., Farber E. (1973). Patterns of damage and repair of liver DNA induced by carcinogenic methylating agents in vivo.. Cancer Res.

[OCR_00778] Gabridge M. G., Denunzio A., Legator M. S. (1969). Cycasin: detection of associated mutagenic activity in vivo.. Science.

[OCR_00789] KISSANE J. M., ROBINS E. (1958). The fluorometric measurement of deoxyribonucleic acid in animal tissues with special reference to the central nervous system.. J Biol Chem.

[OCR_00795] KOBAYASHI A., MATSUMOTO H. (1965). STUDIES ON METHYLAZOXYMETHANOL, THE AGLYCONE OF CYCASIN. ISOLATION, BIOLOGICAL, AND CHEMICAL PROPERTIES.. Arch Biochem Biophys.

[OCR_00800] Kohn K. W., Erickson L. C., Ewig R. A., Friedman C. A. (1976). Fractionation of DNA from mammalian cells by alkaline elution.. Biochemistry.

[OCR_00806] Laqueur G. L., Spatz M. (1968). Toxicology of cycasin.. Cancer Res.

[OCR_00824] McCann J., Ames B. N. (1976). Detection of carcinogens as mutagens in the Salmonella/microsome test: assay of 300 chemicals: discussion.. Proc Natl Acad Sci U S A.

[OCR_00816] McCann J., Choi E., Yamasaki E., Ames B. N. (1975). Detection of carcinogens as mutagens in the Salmonella/microsome test: assay of 300 chemicals.. Proc Natl Acad Sci U S A.

[OCR_00830] Nagata Y., Matsumoto H. (1969). Studies on methylazoxymethanol: methylation of nucleic acids in the fetal rat brain.. Proc Soc Exp Biol Med.

[OCR_00836] Parodi S., Taningher M., Santi L., Cavanna M., Sciaba L., Maura A., Brambilla G. (1978). A practical procedure for testing DNA damage in vivo, proposed for a pre-screening of chemical carcinogens.. Mutat Res.

[OCR_00843] Shank R. C., Magee P. N. (1967). Similarities between the biochemical actions of cycasin and dimethylnitrosamine.. Biochem J.

[OCR_00848] Shibuya C., Hirono I. (1973). Relations between postnatal days of mice and carcinogenic effect of cycasin.. Gan.

[OCR_00863] TERNI M., LO MONACO G. B. (1958). Coltura continua di cellule derivate da embrione umano.. Sperimentale.

[OCR_00868] van den Berg H. W. (1974). Alkaline sucrose gradient sedimentation studies of DNA from HeLa S3 cells exposed to methyl methanesulphonate or methylazoxymethanol acetate.. Biochim Biophys Acta.

